# Pulmonary Manifestations of Inborn Errors of Immunity: Diagnostic and Therapeutic Insights

**DOI:** 10.3390/life15121838

**Published:** 2025-11-29

**Authors:** Katarzyna Napiorkowska-Baran, Szczepan Cofta, Paweł Treichel, Marta Tykwinska, Kinga Lis, Aleksandra Matyja-Bednarczyk, Bartłomiej Szymczak, Maciej Szota, Jozef Slawatycki, Michal Kulakowski, Zbigniew Bartuzi

**Affiliations:** 1 Department of Allergology, Clinical Immunology and Internal Diseases, Collegium Medicum Bydgoszcz, Nicolaus Copernicus University, 87-100 Torun, Poland; knapiorkowska@cm.umk.pl (K.N.-B.); scofta@ump.edu.pl (S.C.); marta.tykwinska@cm.umk.pl (M.T.); kinga.lis@cm.umk.pl (K.L.); maciejszota98@gmail.com (M.S.);; 2 Department of Pulmonology, Allergology and Pulmonary Oncology, Poznan University of Medical Sciences, 61-701 Poznan, Poland; 3 Doctoral School of Medical and Health Sciences, Collegium Medicum Bydgoszcz, Nicolaus Copernicus University, 87-100 Torun, Poland; treichel.pawel@gmail.com (P.T.); slawatyckijozef@gmail.com (J.S.); 4 Center for Transplantology and Interstitial Lung Diseases, Jagiellonian University Medical College, 31-008 Krakow, Poland; matyjabed@gmail.com; 5 Department of Internal Medicine, Jan Biziel University Hospital No. 2 in Bydgoszcz, 85-168 Bydgoszcz, Poland; bartlomiej.szymczak1@gmail.com; 6 Clinical Department of Orthopedics and Traumatology, Collegium Medicum Bydgoszcz, Nicolaus Copernicus University, 87-100 Torun, Poland

**Keywords:** inborn errors of immunity, primary immunodeficiency, pulmonary complications, bronchiectasis, respiratory tract infections, lungs, pneumonia, lung diseases, monitoring

## Abstract

Background: Inborn errors of immunity (IEIs) are a heterogeneous group of genetically determined disorders that lead to immune dysfunction, recurrent infections, and organ-specific complications. The lungs are among the most commonly affected organs, with both infectious and noninfectious manifestations that significantly contribute to morbidity and mortality. This study aimed to provide a comprehensive overview of pulmonary manifestations in IEI, with emphasis on pathophysiological mechanisms, diagnostic approaches, and therapeutic strategies. Methods: A narrative review and synthesis of current literature and clinical guidelines were conducted, focusing on pulmonary involvement in IEI as classified by the International Union of Immunological Societies (IUIS). The analysis included data on infectious and noninfectious complications, imaging findings, immunological assessments, and management strategies, supported by clinical evidence and expert consensus. Results: Pulmonary manifestations in IEI encompass a wide spectrum of conditions. Infectious complications include recurrent bacterial pneumonias, bronchitis, and opportunistic infections, frequently resulting in irreversible lung damage such as bronchiectasis. Noninfectious complications, including granulomatous–lymphocytic interstitial lung disease (GLILD) and interstitial lung disease (ILD), are common in disorders such as common variable immunodeficiency (CVID) and X-linked agammaglobulinemia (XLA). Early diagnosis using high-resolution computed tomography (HRCT) and immunological testing, combined with the timely initiation of immunoglobulin replacement therapy and anti-biotic prophylaxis, significantly improves prognosis. Conclusions: Pulmonary complications are key clinical indicators of IEI and often precede systemic manifestations. Early, integrated, and interdisciplinary diagnostic and therapeutic management are crucial for preventing irreversible lung damage and improving patient outcomes. Regular monitoring and individualized therapy, including immunoglobulin replacement, targeted immunosuppression, and vaccination, are the cornerstones of effective long-term care in IEI.

## 1. Introduction

Immune system disorders, although often perceived as rare and complex to diagnose, have a significant impact on several organ systems, including the respiratory system. Primary immunodeficiencies (PIDs), currently referred to as inborn errors of immunity (IEIs), are a group of genetically determined immune disorders that lead to increased susceptibility to infections and organ complications [1,2]. Pulmonary manifestations are particularly common, often constituting the first or dominant clinical symptom of IEI, and are of fundamental importance in the diagnostic and therapeutic process [1]. Contrary to popular belief, not all immune deficiencies are rare. For instance, selective IgA deficiency has a prevalence of 1 in 142 in the Caucasian population [2].

According to the latest classification by the International Union of Immunological Societies (IUIS), approximately 550 diseases have now been identified as IEIs [3]. Considering the growing recognition of rare diseases and advances in diagnostic methods, the issue of pulmonary complications in primary immunodeficiencies is becoming even more significant in both daily clinical practice and scientific analysis. This article addresses the topic of pulmonary manifestations in patients with inborn errors of immunity, emphasizing the diagnostic aspects and clinical significance of these complications. Since recurrent respiratory infections and non-infectious complications may be pivotal to the early diagnosis of IEI, understanding the underlying pathomechanisms is essential for implementing effective prevention and treatment. Interdisciplinary cooperation is particularly significant in the management of patients with primary immunodeficiencies, as these patients require comprehensive care from a range of medical specialists, including family physicians, clinical immunologists, and pulmonologists [1,4].

Typical and atypical pulmonary manifestations, both infectious (including recurrent pneumonia and bronchiectasis) and noninfectious (chronic interstitial pneumonia, granulomatous–lymphocytic interstitial lung disease (GLILD)), may be substantial indicators of IEI and their early identification improves the prognosis [1,5]. The objective of this study is to provide a comprehensive analysis and overview of pulmonary manifestations of IEIs, including pathophysiological mechanisms, the spectrum of infectious and non-infectious complications, diagnostic methods, and therapeutic options.

## 2. Pathophysiological Mechanisms of Inborn Errors of Immunity

Inborn errors of immunity are a group of genetically determined disorders that result from molecular and cellular defects, leading to dysfunction of the immune system. In recent years, the number of identified genetic defects responsible for these conditions has increased significantly, as the IUIS has systematically updated the IEI classification, enabling a deeper understanding of these disorders. This approach facilitates a more precise diagnosis and more effective management of these diseases, including their severe pulmonary complications [3,6,7]. The classification of IEI into ten main categories, presented in Table 1, is based on the specific clinical and immunological characteristics. This distinction not only facilitates the diagnostic process but also enables the modification of therapeutic strategies, thereby markedly enhancing the prognosis for such patients. Concurrently, this classification expedites the identification of individuals susceptible to severe pulmonary complications, thus emphasizing its clinical significance [3,7].

The disorders can affect any component of the immune system, though, in the majority of cases, they primarily affect humoral immunity (over 50% of cases). Typically, IEIs manifest as infections; however, they may also lead to the development of autoimmune diseases, cancers, or allergies [8,9,10]. The nature of these disorders, in conjunction with the type of pathogen involved in the case of infection, can guide medical professionals toward an accurate diagnosis. The body’s impaired ability to fight pathogens is particularly evident in the respiratory system, which is constantly exposed to microorganisms [1,11]. To further simplify the diagnosis of IEIs, the Jeffrey Modell Foundation has developed a set of ten warning signs in children and adults that may indicate a primary immunodeficiency (Table 2). The importance of age differentiation is predicated on the fact that the immune system in children is still maturing. Additionally, exposure to crowded environments, such as nurseries or kindergartens, may also have an impact [12,13]. 

**Table 2:** The Jeffrey Modell Foundation’s 10 warning signs of primary immune deficiency [12].

**WARNING SIGNS IN CHILDREN**
1. ≥4 new ear infections within 1 year
2. ≥2 serious sinus infections within 1 year
3. ≥2 months on antibiotics with little effect
4. ≥2 cases of pneumonia within 1 year
5. Failure of an infant to gain weight or grow normally
6. Recurrent, deep skin, or organ abscesses
7. Persistent thrush in mouth or fungal infection on skin
8. Need for intravenous antibiotics to clear infections
9. ≥2 deep-seated infections including septicemia
10. A family history of PID
**WARNING SIGNS IN ADULTS**
1. ≥2 new ear infections within 1 year
2. ≥2 new sinus infections within 1 year, in the absence of allergy.
3. 1 case of pneumonia per year for >1 year
4. Chronic diarrhea with weight loss
5. Recurrent viral infections (colds, herpes, warts, condyloma)
6. Recurrent need for intravenous antibiotics to clear infections.
7. Recurrent, deep abscesses of the skin or internal organs
8. Persistent thrush or fungal infection on skin or elsewhere
9. Infection with normally harmless tuberculosis-like bacteria
10. A family history of PID

Immune deficiencies affecting both cellular and humoral immunity are severe disorders characterized by defects in both T and B lymphocytes [14].

T cells are central mediators of adaptive immune responses and play a critical role in maintaining pulmonary immune homeostasis. They mature in the thymus and are divided into distinct subsets, including CD4+ helper, CD8+ cytotoxic, regulatory Treg and memory T cells, each of which contribute uniquely to immune defense and tissue repair. In lungs, T-cells response against viral and bacterial pathogens such as *Mycobacterium* spp. regulates inflammation and supports epithelial regeneration following injury. Impaired T-cell function, however, increases susceptibility to frequent pulmonary infections, highlighting their essential role in maintaining respiratory immune defense [15,16,17].

A prime example is severe combined immunodeficiency (SCID), which presents with severe, recurrent infections during the first months of life. The patient’s condition necessitates urgent medical attention, which may include hematopoietic stem cell transplantation [18]. Combined immunodeficiencies with syndromic features include diseases in which immunodeficiency coexists with malformations of other systems. DiGeorge syndrome (22q11.2), for instance, is characterized by dysfunction of the cellular immune system and the presence of congenital anomalies in the heart, face, and palate [19,20].

Predominantly, antibody deficiencies are the most prevalent group of primary immunodeficiencies. These include, among others, selective IgA deficiency, CVID (common variable immunodeficiency), and IgG subclass deficiency, and manifest themselves as recurrent respiratory tract infections, inadequate response to vaccinations, and, in some cases, the development of autoimmunity. An intriguing deficiency in this group is the lack of specific antibodies. This immune disorder is characterized by normal levels of total immunoglobulins (IgG, IgA, and IgM), yet an inability to produce effective antibodies in response to infections or vaccinations, particularly against polysaccharide antigens (e.g., *Streptococcus pneumoniae*) [21,22].

Disorders associated with immune system dysregulation include conditions in which the immune system is unable to adequately regulate its own activity, leading to a range of severe outcomes, including autoimmunity (the body’s own tissues are attacked), lymphoproliferation, or immunodeficiency. Examples of such conditions include immunodysregulation polyendocrinopathy enteropathy X-linked syndrome (IPEX) and autoimmune lymphoproliferative syndrome (ALPS) [23,24]. Congenital defects in the number or function of phagocytes refer to an abnormal number or function of phagocytic cells (neutrophils, macrophages), resulting in increased susceptibility to bacterial and fungal infections, such as chronic granulomatous disease (CGD) and congenital neutropenia [25,26]. Defects in intrinsic and innate immunity are disorders that affect the mechanisms of rapid, nonspecific immune responses, including interferon signaling. They often manifest as severe viral and bacterial infections. Examples include TLR, IRF7, and IFNAR deficiencies [27,28,29].

Autoinflammatory disorders are characterized by the uncontrolled activation of the innate immune system, leading to recurrent inflammation that is typically devoid of autoantibodies. The manifestations of this condition include recurrent fevers, arthritis, and skin lesions. Representative examples of this group include familial Mediterranean fever (FMF), TNF receptor-associated periodic syndrome (TRAPS), and neonatal-onset multisystem inflammatory disease (NOMID) [30]. Complement system deficiencies encompass deficiencies in complement proteins, which result in increased susceptibility to infections, especially those caused by *Encapsulated bacteria*, and a propensity for autoimmune disorders. Examples of such mechanisms include C2, C3, and C5 deficiencies [31]. Bone marrow failure refers to disorders that result in a reduced production of blood cells, including immune cells. Examples include Fanconi anemia and Shwachman–Diamond syndrome (SDS) [32]. Phenocopies of inborn errors of immunity are conditions that clinically resemble primary immunodeficiencies but are not caused by hereditary mutations [33].

## 3. The Spectrum of Pulmonary Manifestations

Pulmonary manifestations in patients with inborn errors of immunity include a variety of infectious and noninfectious complications that substantially affect their quality of life. It is crucial to recognize and monitor these changes in order to effectively implement appropriate therapeutic strategies.

### 3.1. Infectious Complications

Respiratory infections are a key challenge for patients with IEI, although recurrent pneumonia, bronchitis, and otitis media are frequently both the first and most prominent clinical manifestations of these disorders. Repeated infections lead to chronic local inflammation which may cause permanent structural lung damage, such as bronchiectasis or chronic bronchitis. Cohort studies confirm that recurrent respiratory tract infections contribute to irreversible changes in lung structure, even in the early stages of the disease, which significantly affects patients’ quality of life and increases their susceptibility to further complications [5,34].

The variability of infectious complications, including the aforementioned changes, depends strictly on the type of immunodeficiency, implying the necessity for precise differentiation at the diagnostic stage [35]. In antibody-deficient individuals, sinopulmonary complications are observed more commonly, requiring particular clinical vigilance and an interdisciplinary approach [35,36].

The characteristics of infectious agents in IEI include both typical pathogens, such as *Streptococcus pneumoniae* and *Haemophilus influenzae*, and opportunistic microorganisms, including *Pneumocystis jirovecii*, cytomegalovirus (CMV), and *Mycobacterium* avium complex (MAC). These agents not only cause severe infections but also often exhibit resistance to standard treatment, requiring individually tailored therapeutic regimens [35,37]. The high incidence of *Streptococcus pneumoniae* and *Haemophilus influenzae* infections leads to chronic inflammatory changes in lung tissue; in turn, delayed diagnosis may contribute to the development of irreversible structural lung complications [5,38]. Opportunistic microorganisms, such as *Pneumocystis jirovecii* or CMV, particularly in patients with severe cellular immunodeficiency, have a very serious clinical course, necessitating hospitalization and combined therapy [37,39]. Regarding other atypical pathogens, such as *Mycobacterium* spp., Salmonella, and Shigella, they tend to cause systemic infections, posing an additional diagnostic and therapeutic challenge [37,40,41]. Viral infections, including those caused by herpesviruses or enteroviruses, are characterized by a more severe course in affected individuals, necessitating meticulous monitoring and prompt initiation of suitable treatment [37]. In the case of multifactorial infections, it is essential to consider the complex clinical picture, which underscores the importance of specialized diagnostic methods, such as microbiological and molecular testing, to identify the etiological agent [35].

Despite the implementation of standard treatment methods such as immunoglobulin substitution and prophylactic antibiotic therapy, patients with IEI remain vulnerable to chronic and recurrent respiratory infections. Although these therapeutic interventions have been proven to reduce the severity and frequency of infections, they do not completely eliminate the risk of developing chronic structural lung complications, such as bronchiectasis or chronic obstructive pulmonary disease. It should be noted that the frequency and severity of infectious complications in patients with IEI vary considerably, contingent on age and the specific clinical disorder [5,35].

The relationship between inborn errors of immunity and the etiological factors of infections is demonstrated in Table 3.

### 3.2. Noninfectious Complications

Noninfectious pulmonary complications in patients with IEI present a substantial clinical challenge requiring a comprehensive diagnostic and therapeutic approach. Bronchiectasis, as a chronic non-infectious complication, is particularly common in people with common variable immunodeficiency (CVID) and X-linked agammaglobulinemia (XLA). These conditions result from chronic inflammation and recurrent infections, leading to the destruction of the respiratory tract epithelium. Research indicates that as many as 61% of patients with CVID and 47% of individuals with XLA under the age of 50 are diagnosed with bronchiectasis [42,43,44,45]. Despite the implementation of adequate therapeutic interventions, such as immunoglobulin substitution or antibiotic therapy, bronchiectasis remains a challenging condition to treat, underscoring the need for specialized care in the early diagnosis and prevention of these alterations [42,44].

Interstitial lung diseases (ILDs), including granulomatous lymphocytic interstitial lung disease (GLILD) and lymphoid interstitial pneumonia (LIP), constitute additional substantial non-infectious complications associated with CVID. These conditions, which occur in 10–20% of patients, are characterized by clinical and imaging findings that confirm the presence of lymphoid hyperplasia, granulomas, and chronic inflammation [42,44,46]. These lesions have the potential to result in the development of pulmonary fibrosis and impaired respiratory function. On high-resolution computed tomography (HRCT) imaging, GLILD is characterized by lymphoid infiltrates, non-caseating granulomas, and organizing pneumonia, as confirmed by subsequent histopathological findings [42,44]. Clinical symptoms of such conditions include chronic cough, shortness of breath, fever, and weight loss; however, the disease may sometimes be mildly symptomatic or asymptomatic, which hinders early diagnosis [46]. Interstitial lung abnormalities lead to significant deterioration in respiratory function and increase the risk of developing lymphoma [44]. Recent studies have demonstrated that GLILD is frequently underreported in the CVID population. This is due to the fact that these manifestations are detected incidentally during imaging performed for other reasons, which highlights the need for active screening for these complications in at-risk patients [42].

Non-infectious lung lesions in hyper-IgE syndrome (HIES) and chronic granulomatous disease (CGD) are of particular significance, as they can lead to permanent damage, such as pneumatoceles, bronchiectasis, and mosaic pulmonary perfusion, posing a significant clinical challenge. In HIES, these lesions affect up to 75% of patients and result from chronic inflammation, autoimmune reactions, and abnormal tissue repair [42,43,46]. Distinctive features of HIES include the presence of large, thin-walled air cavities (pneumatoceles), which predispose patients to bacterial infections and chronic pulmonary aspergillosis [43,46]. In CGD, scarring and remodeling of the lung architecture predominate, leading to mixed functional disorders and the occurrence of granulomas and fibrosis [46,47]. The lesions of this kind often result in chronic respiratory failure and, in severe cases, may require lung transplantation [46].

Noninfectious pulmonary complications in children with primary immunodeficiencies may be asymptomatic, and the presence of changes in the lung parenchyma is often detected incidentally during imaging studies such as HRCT. Regular monitoring of lung status is crucial for the early detection of these changes and the implementation of appropriate treatment, thereby reducing the risk of irreversible damage [43,46]. The immaturity of the immune system and the frequent undiagnosed early stages of the disease further increase the risk of complications in children. Delays in diagnosis and treatment can lead to permanent lung damage before pronounced clinical symptoms appear [46]. The regular use of advanced diagnostic techniques, such as HRCT and pulmonary function tests, enables early detection of even subtle structural changes, supporting the treatment process and limiting disease progression [43,48]. The absence or scarcity of symptoms should not exclude children from active monitoring, as delays in therapeutic intervention may result in an unfavorable prognosis [46].

A delayed diagnosis and a lack of appropriate treatment are pivotal factors contributing to the progression of non-infectious complications in patients with primary immunodeficiencies. Conditions such as bronchiectasis, ILD, and GLILD are significantly associated with the severity of immunodeficiency and delayed immunoglobulin therapy [44,49]. It is estimated that over 70% of patients with CVID develop permanent structural lung changes before being diagnosed with IEI, which unequivocally indicates the need for early detection of these disorders [49]. The clinical effects of such complications include chronic cough, shortness of breath, recurrent infectious exacerbations, and progressive deterioration of respiratory function, resulting in significant limitations on daily activities [44]. Early identification and immunoglobulin treatment may reduce the extent of permanent impairment and improve prognosis; therefore, it is justified to implement appropriate screening programs and educate both patients and their families [49,50]. Close interdisciplinary collaboration, involving immunologists, pulmonologists, pediatricians, and family physicians, is essential for the early detection, optimization of treatment, and prevention of pulmonary complications in primary immunodeficiencies [44,51,52].

Given that pulmonary manifestations of IEIs, both infectious and non-infectious, often coexist, Table 4 presents a summary of symptoms in alphabetical order, intended as a practical aid for those involved in the care of patients with these disorders. The table was developed based on the 2024 IEI classification [3].

## 4. Diagnostics and Medical Monitoring

Diagnostics and medical monitoring are critical components of effective patient care for individuals with IEI, as they facilitate early detection of complications and optimize treatment. Among the diagnostic techniques employed, advanced imaging methods and immunological assessments are of particular significance, as collectively they provide a comprehensive overview of the patient’s health status. Adequate monitoring is crucial for minimizing the risk of permanent respiratory damage and enhancing patient quality of life [48,53,54].

### 4.1. Medical Imaging

Imaging studies of the lungs are instrumental in diagnosing and monitoring pulmonary complications in patients with primary immunodeficiencies, enabling the precise detection of structural changes such as bronchiectasis, bronchial wall thickening, mucus plugging, and mosaic perfusion. According to studies, these changes affect up to 80% of patients with primary immunodeficiencies [42,48]. High-resolution computed tomography is a fundamental diagnostic tool that facilitates the differentiation between infectious and non-infectious complications and the identification of chronic inflammatory changes, which, in turn, enables the expeditious implementation of targeted treatment to limit the progression of lung parenchymal damage [42,46,48]. It is noteworthy that HRCT is more sensitive than conventional X-rays, enabling the detection of minor structural changes in the early stages of disease development, even in asymptomatic cases [46,55]. This kind of precise diagnostic approach is extremely important, especially in pediatric populations, where clinical symptoms may be subtle and structural changes progress imperceptibly.

The characteristics of the radiological image vary depending on the disease entity. In cases of CVID, the most common pathologic findings are bronchiectasis and thickening of the bronchial walls. In contrast, patients with chronic granulomatous disease typically exhibit consolidation and mosaic perfusion. In patients with HIES, pneumatoceles and chronic fibrotic changes are reported [42,56]. A thorough analysis of the extent and nature of imaging changes not only facilitates a better understanding of the disease’s dynamics but also enables the assessment of the risk of progression of pulmonary complications. In some cases, this analysis influences therapeutic decisions, such as intensifying immunosuppression in GLILD or changing the antibiotic therapy strategy in situations involving massive mucus plugging. Another salient aspect pertains to the capacity for continuous monitoring of treatment effects and the progression of complications, which allows for ongoing optimization of therapy [5,42].

HRCT is considered the “gold standard” in assessing the severity of bronchiectasis and other chronic pulmonary complications, as it detects changes even in patients without evident clinical symptoms. Therefore, the early implementation of therapeutic interventions, such as respiratory physiotherapy or chronic antibiotic therapy, is feasible [1,46]. Furthermore, HRCT enables the precise localization and assessment of lesion extent, including air trapping, which is prognostically significant and influences the choice of therapeutic measures [42]. In patients with GLILD, this technique is invaluable for detecting parenchymal interstitial lesions, which require special consideration when selecting immunosuppressive therapy and monitoring health status [42,46].

The ability to detect lesions that evade conventional radiological assessments holds particular diagnostic significance, especially in pediatric cases, where the clinical manifestation is often asymptomatic. Proactive diagnostic imaging facilitates immediate treatment adjustment, thereby reducing the risk of irreversible effects, such as chronic lung damage [46]. According to international guidelines, lung conditions should be routinely monitored, with HRCT performed at intervals of 3 to 5 years. This approach facilitates the identification of progressive changes, the evaluation of treatment efficacy, and the early detection of complications [5]. Although this examination schedule is widely accepted, it is occasionally necessary to adjust the frequency of examinations to the individual risk of disease progression or the altered clinical condition of the patient, particularly in cases of primary immunodeficiencies that are dynamically progressing [42].

The implementation of routine imaging tests also brings benefits in terms of assessing the effectiveness of therapy. For instance, regular HRCT scans can provide evidence of a reduction in the number of new infectious foci or a slowdown in the progression of structural changes in the lungs, which promotes the optimization of treatment, including immunoglobulin therapy [1,5]. Nonetheless, the analysis of such tests should be compared with information from the interview, laboratory results, and a detailed clinical picture in order to rule out other potential causes of chronic lung changes, such as cystic fibrosis, chronic obstructive pulmonary disease (COPD), or chronic aspiration [57].

It is essential to recognize that X-rays and CT scans are contraindicated in individuals with immunodeficiencies that increase radiosensitivity. The only exception should be life-threatening conditions. Ultrasound or magnetic resonance imaging is recommended as an alternative. PIDs associated with radiosensitivity include: SCID with hypersensitivity to ionizing radiation and deficiencies associated with DNA repair disorders, such as ataxia-telangiectasia syndrome, Nijmegen syndrome, and RIDDLE syndrome [58,59,60].

Given the high prevalence and often silent progression of these pulmonary changes, a structured and continuous monitoring strategy remains essential for optimal long-term management in patients with IEIs. Monitoring pulmonary involvement in patients with IEIs plays a key role in the early detection of complications that may significantly influence disease progression and quality of life. In many patients, particularly those with antibody deficiencies, chronic and recurrent respiratory infections can lead to irreversible structural lung damage, including bronchiectasis or fibrotic remodeling, which may already be present at the time of diagnosis [48,61]. Routine functional assessment, such as spirometry or gas transfer evaluation, performed alongside periodic imaging studies, enables early recognition of pathological changes and timely therapeutic intervention, including optimization of immunoglobulin replacement, respiratory physiotherapy, or the introduction of prophylactic antibiotic regimens. Effective monitoring and prevention of progressive lung disease represent a key component of comprehensive care for patients with IEIs, contributing to improved long-term outcomes and quality of life [62,63,64].

### 4.2. Immunological Assessment

The assessment of immunological parameters is a critical step in the diagnostic process for patients with primary immunodeficiency disorders. The evaluation of immunoglobulin concentrations, such as IgG, IgA, and IgM, as well as IgG subclasses, provides valuable information about the nature of the immunodeficiency. For instance, decreased IgG and IgA concentrations with normal or elevated IgM may indicate CVID or hyper-IgM syndrome, while a complete absence of immunoglobulins and B lymphocytes suggests X-linked agammaglobulinemia [65,66,67,68]. However, caution is advised when interpreting these results, as differences in immunoglobulin concentrations may be attributable to factors such as age or the concomitant presence of other diseases, which highlights the need for a comprehensive clinical picture [69,70]. In instances where the measurement of immunoglobulin concentrations is not feasible, a proteinogram can be used as a preliminary diagnostic tool, due to the fact that immunoglobulins are mainly present in the γ-globulin fraction (all immunoglobulin classes) and to a lesser extent in the β-globulin fraction (IgA and IgM) [71]. An alternative, inexpensive test is the calculation of globulin, which is determined by subtracting the total protein value from the albumin value. In studies conducted by Jolles, a cutoff value of <18 g/L for calculated globulin was determined to define a population in which 89% had an IgG concentration of <6 g/L [72].

A precise assessment of lymphocyte subpopulations, including CD3, CD4, CD8, CD19, and CD16/56, enables the identification of cellular immune disorders characteristic of conditions such as complex immunodeficiencies. The analysis of these parameters not only enables diagnosis, but also monitoring of the dynamics of immune changes in response to therapy [73,74,75]. For instance, the number of CD4 lymphocytes may be of prognostic significance, particularly in the context of the risk of opportunistic infections [76]. However, the interpretation of these data should take into account potential confounding factors, such as immunosuppressive therapy or concomitant viral infections [7].

Functional tests, such as evaluating lymphocyte response to mitogens or examining specific post-vaccination responses, are indispensable in detecting subclinical defects in humoral responses. The absence of an increase in antibody levels subsequent to vaccination against *Streptococcus pneumoniae* or *Haemophilus influenzae*, for instance, may indicate the presence of selective antibody production defects in patients with recurrent lung infections [77,78,79]. These tests are a particularly valuable diagnostic tool in patients with inconclusive results from basic immunological tests, enabling more precise differentiation between primary and secondary immunodeficiencies [77,78].

In addition antigen-specific T-cell responses can be assessed using interferon-gamma release assays (IGRAs) or ELISpot techniques, which measure cytokine produc-tion following stimulation with microbial or recall antigens such as tuberculosis or cy-tomegalovirus. They can affect lungs and cause inflammation especially patients with immunodeficiencies who are prone to them [80].

B-cell functions is most often assessed indirectly through measurement of immuno-globulin levels and evaluation of specific antibody responses following immunization with polysaccharides or peptide antigens. These assays rely on serum or plasma samples and often require pre- and post-immunization measurements to evaluate functional capacity [81,82].

The interpretation of immunological test results should take into account the patient’s age, as the reference values for immunoglobulins and lymphocytes change during a child’s development. For instance, a physiological decrease in IgG concentration in newborns, which results from the disappearance of maternal antibodies, or transient neutropenia in infants [83,84]. Nevertheless, persistent deviations require in-depth diagnostics to rule out primary immunodeficiencies. Delays in assessing such abnormalities can lead to serious health consequences, underscoring the critical importance of early diagnosis [85].

Concurrent interpretation of immunological and microbiological diagnostic results is crucial for differentiating between primary and secondary immunodeficiencies and ruling out other causes of recurrent lung infections, such as cystic fibrosis, chronic granulomatous disease, or primary ciliary dyskinesia. An integrated diagnostic approach has been shown to reduce the risk of misdiagnosis and expedite the implementation of appropriate therapy. One such example is the analysis of the immune response to bacterial or viral pathogens, which enables the differentiation between primary and secondary infections [52,85].

Modern technologies, such as next-generation sequencing (NGS), represent a breakthrough in the genetic diagnosis of IEI, enabling the detection of single gene mutations responsible for specific subtypes of the disease [86]. A prime example is the identification of mutations in the BTK gene in patients with XLA or mutations in the CTLA-4, LRBA, or GATA2 genes in patients with immunoregulatory defects [87,88,89,90]. The implementation of genetic testing not only facilitates definitive diagnostic confirmation but also allows for predicting the risk of developing pulmonary complications and planning preventive measures. However, genetic test results must always be interpreted in the context of the patient’s clinical phenotype and immune response, as even the same mutation can lead to a variety of clinical manifestations [7,11].

In clinical practice, the assessment of lymphocyte subpopulations and other immunological tests is performed using whole blood samples, with analysis conducted, for example, by flow cytometry [91]. The CD3 marker reflects the total T lymphocyte count, while the CD4 and CD8 subpopulations allow for the evaluation of the proportions of helper and cytotoxic T cells. CD19 serves as a marker for B lymphocytes, and CD16/56 identifies the natural killer (NK) cell population [92,93]. These results are of key importance in the diagnosis of primary immunodeficiencies. For example, a markedly reduced number of B lymphocytes (CD19+) with a normal T lymphocyte count suggests agammaglobulinemia, whereas a reduction in both T (CD3+) and B (CD19+) cells is characteristic of severe combined immunodeficiency (SCID) [94,95,96]. A decreased CD4 lymphocyte count may also have prognostic significance and is associated with an increased risk of opportunistic infections [97]. Interpretation of these parameters should take into account the patient’s age, clinical condition, and any ongoing immunosuppressive therapy.

Immunological assessment is a fundamental element in the diagnosis and monitoring of patients with IEIs. Table 5 presents, among other things, tests that are helpful in diagnosing specific types of immune deficiencies and monitoring pulmonary complications in patients with IEIs.

## 5. Therapeutic Management

The primary treatment for patients with antibody deficiencies, such as CVID and XLA, is immunoglobulin replacement therapy, which significantly reduces the frequency of lung infections, limits the risk of developing bronchiectasis, and reduces the long-term structural complications of the lungs. The therapy involves the administration of immunoglobulins intravenously (IVIG) or subcutaneously (SCIG), which increases antibody concentrations and augments the patient’s immune mechanisms [99,100,101]. A number of studies document the effectiveness of this approach; however, the necessity of individualizing dosages and adjusting treatment regimens to the needs of specific patients is also emphasized, particularly in the context of the dynamics of the clinical course of the disease and treatment response indicators [101,102,103]. It should be noted that the patient receives IgG antibodies; therefore, this therapy is not applicable in cases of selective IgA or IgM deficiency, among others. In addition, immunoglobulin preparations contain a mixture of antibodies derived from a minimum of 1000 donors to ensure the best possible efficacy [104,105]. This has implications for antibody-based diagnostics, as the presence of antibodies from immunoglobulin donors may result in false-positive test results. Therefore, this type of diagnostic evaluation should be conducted a minimum of six weeks subsequent to the most recent immunoglobulin transfusion, or alternative methods should be employed. Notably, the initial IgG concentration is not a criterion for the inclusion of immunoglobulin preparations, but depends on the clinical condition, particularly the frequency and severity of infections [106,107].

The European Society for Immunodeficiencies (ESID) recommends the following approach to determine when IgGRT administration is indicated for serum immunoglobulin reconstitution:

• IgG < 200 mg/dL: All patients (except children, who may have physiological hypogammaglobulinemia without severe infections);
• IgG levels 200–500 mg/dL: When deficiency is identified and associated with recurrent infections;
• IgG > 500 mg/dL: When there is a deficiency in the production of antibodies against antigens and severe or recurrent infections [108].

Antibiotic prophylaxis and rapid, targeted antibiotic therapy are both essential parts of treating patients with recurrent lower respiratory tract infections caused by IEIs. Research studies indicate that the use of antibiotics for prophylaxis significantly reduces the risk of developing structural lung damage, such as bronchiectasis, as well as the number of serious infections and hospitalizations [35,61]. However, these protocols must be individualized, taking into account local microbial resistance profiles and the patient’s history of infections. It is necessary to balance the benefits of antibiotic prophylaxis with the risk of developing bacterial resistance, which may require interdisciplinary collaboration in the development of therapeutic strategies [61,109,110,111].

**Figure 1:**
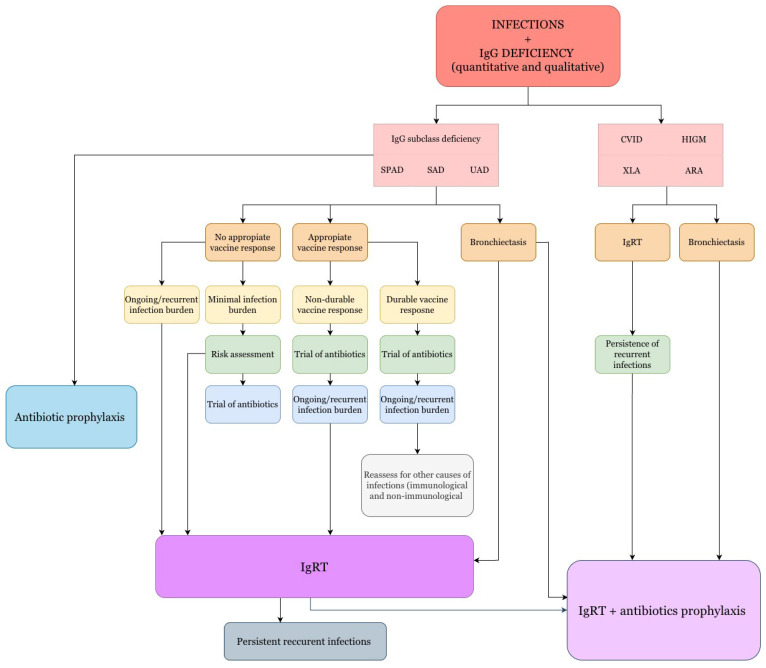
Therapeutic management algorithm for recurrent infections in patients with antibody production disorders, with or without accompanying pulmonary changes, such as bronchiectasis [95,99].

The therapeutic algorithm for recurrent infections in patients with antibody production disorders, with or without accompanying pulmonary changes such as bronchiectasis, is presented in Figure 1. In selected clinical cases, the presence of additional structural changes in the lungs should also be considered as a factor supporting the implementation of immunoglobulin therapy.

In summary, ongoing antibacterial prophylaxis is recommended for patients with chronic granulomatous disease (CGD), hyper-IgE syndrome, and selected PIDs associated with neutropenia, severe, chronic, or recurrent infections, as well as in the presence of complications such as bronchiectasis. Some patients require periodic prophylaxis, typically lasting 3–6 months, most often during the fall and winter months or in situations with an increased risk of infection (e.g., after tooth extraction, ENT, or orthopedic procedures). In such cases, antibiotic therapy is typically administered for 5–7 days after the procedure. Extended antibiotic therapy may also be necessary for recurrent or chronic infections, such as sinusitis, otitis media, pneumonia, or urinary tract infections (UTIs). The choice of antibiotic depends on the etiology of the microorganism: in infections caused by *Staphylococcus* aureus, trimethoprim/sulfamethoxazole is most often used, while in infections caused by Streptococcus spp., Mycoplasma spp., or nontuberculous mycobacteria (NTM), azithromycin is the antibiotic of choice [112].

Ongoing antifungal prophylaxis is indicated in patients with chronic granulomatous disease, hyper-IgE syndrome, and chronic mucocutaneous candidiasis. The choice of antifungal drug depends on the type of microorganism; itraconazole is typically used for Aspergillus infections, while fluconazole is used for *Candida* infections. Prevention of *Pneumocystis jirovecii* infections is also a crucial element of prophylactic treatment for many patients with IEIs, which is achieved through the long-term use of trimethoprim-sulfamethoxazole (TMP-SMX). Some patients require long-term antifungal therapy, especially in severe forms of infections such as fungal pneumonia or central nervous system mycosis [113,114,115].

Ongoing antiviral prophylaxis is recommended for patients with severe forms of IEIs, such as SCID or DOCK8 deficiency. In some cases, especially in patients with neutropenia or lymphopenia, temporary antiviral therapy is necessary to prevent infections caused by herpes simplex virus (HSV), varicella-zoster virus (VZV), and cytomegalovirus (CMV). Depending on the pathogen, acyclovir is used to treat HSV and VZV infections, while ganciclovir is used to treat CMV infections [116,117].

In selected cases, immunomodulatory therapy, such as interferon gamma, is used in patients with chronic granulomatous disease [112].

The treatment of non-infectious pulmonary complications, such as GLILD in CVID, poses a particular clinical challenge. Immunosuppressive therapy, including glucocorticosteroids and immunomodulatory medications such as rituximab, is effective in stabilizing these changes, but requires careful assessment of the risk of adverse effects, especially secondary infections [118,119,120]. Interdisciplinary cooperation between immunologists and pulmonologists is crucial in this regard, as it enables the precise tailoring of treatment to the patient’s individual needs [42,119,120]. The monitoring of treatment efficacy should be based on regular imaging and pulmonary function tests, which allow for the assessment of parenchymal lesion regression and the management of potential adverse effects. Despite the notable benefits offered by current therapeutic strategies, further research is necessary to develop new immunomodulatory drugs that achieve equivalent efficacy while reducing the risk of adverse effects [11,119,120].

Continuous observation of patients with IEI is a pivotal element of effective therapy. Regular HRCT examinations have been shown to facilitate the detection of progression in changes, such as bronchiectasis or pulmonary parenchymal diseases, even in patients who do not exhibit evident clinical symptoms [42,121,122]. Moreover, functional assessment of the lungs and periodic analysis of immune parameters enable a faster response to deterioration in patients’ health, which is crucial in preventing chronic damage to the respiratory system. Current guidelines suggest the need for integrated monitoring, which enables the individualization of therapeutic strategies based on the patient’s evolving clinical condition [11,121,122]. The regular assessment of treatment efficacy and safety, in conjunction with the dynamic adjustment of therapeutic regimens, is essential for the optimization of patient care [121,122,123].

Protective immunization is a fundamental element of infection prevention in patients with IEI. Inactivated vaccines are considered safe and effective in this patient group, allowing for a reduction in the amount of infections and limiting the progression of pulmonary complications. These can be used in most patients with IEIs. It is recommended to get vaccinated against influenza (annually), pneumococcal disease (PCV13 + PPSV23 or, optionally, PCV20 only), meningococcal (ACWY and B), pertussis (Tdap)—recommended every 10 years—COVID-19 (according to the national program), hepatitis A and B, HPV—especially in adolescents and women, as well as polio (inactivated form—IPV). It is worth noting that in patients with antibody production disorders, vaccinations are effective due to preserved cellular immunity [124,125,126,127]. However, the administration of live vaccines should be considered in limited cases and only after a thorough risk assessment, as in some circumstances it may lead to significant infectious complications [41,124,128]. Contraindications to these vaccinations include severe cellular immune disorders and the use of immunoglobulins. Not to forget about “cocooning” vaccinations, which is a strategy for protecting individuals, particularly vulnerable to severe infectious diseases, by vaccinating those in the immediate environment of the sick or at risk. Education for patients and their caregivers is also of crucial importance in the context of an immunization strategy, playing a key role in increasing compliance with the vaccination program and improving health outcomes [124,128,129].

The optimization of therapeutic approaches in patients with IEI necessitates the involvement of interdisciplinary teams comprising specialists, including immunologists, pulmonologists, pediatricians, and infectious disease experts. Such cooperation facilitates comprehensive diagnosis, effective treatment, and patient monitoring, thereby minimizing the risk of disease progression and improving quality of life [130,131]. The education of patients and their families is an integral component of care, facilitating a more efficient response to infectious symptoms and the long-term maintenance of the optimal quality of life in the course of these rare chronic diseases [132,133,134]. The collaborative efforts of medical professionals, combined with the implementation of novel diagnostic and therapeutic technologies, can significantly contribute to improved treatment outcomes and enhanced patient prognoses [132,133].

Therapeutic strategies targeting T cells in inborn errors of immunity aim to restore immune homeostasis by correcting specific functional defects. Approaches include cytokine modulation, such as low-dose IL-2 to expand regulatory T cells, and JAK inhibitors to normalize aberrant signaling. Costimulatory and checkpoint modulation, exemplified by CTLA-4-Ig and experimental PD-1/PD-L1 interventions, seeks to control hyperactive or exhausted T cells. Gene therapy and gene editing offer the potential to correct underlying genetic defects, while adoptive cell transfer provides functional T cells to restore immune competence [135].

Collectively, these interventions highlight a precision-medicine approach to managing T cell-related immune dysfunction [136].

Despite their promise, T cell-targeted therapies face significant limitations. Many approaches, including cytokine modulation and checkpoint inhibition, carry risks of off-target immune activation or excessive immunosuppression. Moreover, gene therapy and adoptive cell transfer remain experimentally complex, costly, and may be constrained by long-term safety and accessibility concerns [135].

## 6. Summary

Pulmonary manifestations represent one of the earliest and most prominent clinical indicators of inborn errors of immunity, and their presence has significant prognostic value. Early diagnosis of the deficiency is crucial, as it largely determines its course. Diagnostic tools, especially high-resolution computed tomography (unless contraindicated), facilitate early detection of even subclinical complications and rapid implementation of individualized treatment strategies. However, it should be borne in mind that the principle of least harm applies. Consequently, alternative methods should be considered beforehand, such as lung ultrasound, which holds particular significance in pediatric cases. The diagnosis and monitoring of patients should also be based on other available diagnostic methods, such as blood gas analysis, bronchoscopy, and microbiological tests. When considering patients with humoral immune disorders, it is essential to recognize that methods involving antibody testing may not be entirely diagnostic. In the context of immunoglobulin replacement therapy, IgG antibodies detected during testing may actually be antibodies from the immunoglobulin donors. Although genetic testing is very informative, it often does not alter the management of patients in terms of treatment and monitoring.

Despite the implementation of standard therapies, such as immunoglobulin substitution and antibiotic prophylaxis, patients remain at risk of chronic and progressive lung damage, which highlights the necessity for regular, integrated monitoring and flexible adjustment of therapeutic management. Early protective immunization and education of patients and their families as key elements of prevention and quality of life improvement should also not be overlooked.

## Figures and Tables

**Table 1:** Classification of IEIs according to IUIS [3].

Immunodeficiencies affecting cellular and humoral immunity
2. Combined immunodeficiencies with associated or syndromic features
3. Predominantly antibody deficiencies
4. Diseases of immune dysregulation
5. Congenital defects of phagocyte number or function
6. Defects in intrinsic and innate immunity
7. Autoinflammatory disorders
8. Complement deficiencies
9. Bone marrow failure
10. Phenocopies of inborn errors of immunity

**Table 3:** The relationship between inborn errors of immunity (IEI) and typical etiological factors of infections [3,5,7]. Abbreviations: CMV—Cytomegalovirus; EBV—Epstein–Barr Virus; HLH—Hemophagocytic Lymphohistiocytosis; HSV—Herpes Simplex Virus; IBD—Inflammatory Bowel Disease; IEI—Inborn Errors of Immunity; IFN-I—Type I Interferon; IgG/IgA/IgM—Immunoglobulin G/A/M; NK—Natural Killer cells; TLR—Toll-Like Receptor; VZV—Varicella Zoster Virus.

Type of Immunodeficiency	Key Cells/Systems Affected	Typical Pathogens	Main Pulmonary Manifestations
Humoral (antibody-related)	B lymphocytes, antibodies (IgG, IgA, IgM)	*Encapsulated bacteria*: *Haemophilus influenzae*, *Streptococcus pneumoniae*; Viruses: Enteroviruses	*Recurrent otitis*, sinusitis, bronchitis, pneumonia; gastrointestinal infections
Cellular (T-cell)	T lymphocytes	*Opportunistic bacteria*: *Pseudomonas aeruginosa*, *Klebsiella pneumoniae*, *Acinetobacter baumannii*, *Staphylococcus* spp., *Mycobacterium* spp., *Listeria* spp. Viruses: CMV, HSV, VZV; Fungi: *Candida* spp., *Pneumocystis jirovecii*	Disseminated viral and fungal infections, *Opportunistic bacterial* infections, chronic diarrhea, growth failure
Phagocytic defects	Neutrophils, macrophages	*Staphylococcus aureus*, *Serratia marcescens*, *Aspergillus* spp.	Skin/soft-tissue abscesses, liver and lung infections
Complement deficiency—terminal pathway (C5–C9)	Terminal complement components	*Neisseria meningitidis*	*Recurrent meningitis*, sepsis
Complement deficiency—classical pathway (C1q, C2, C4)	Classical complement proteins	S. pneumoniae, H. influenzae	Recurrent respiratory infections
Complement deficiency—C3	Central element of all pathways	*S. pneumoniae*, *H. influenzae*	Severe purulent infections
Defects in intrinsic/innate immunity	Neutrophils, macrophages, NK cells, TLR signaling, IFN-I pathway	*Bacteria*: pyogenic bacteria (streptococci, staphylococci), mycobacteria (M. bovis BCG, atypical), Salmonella Viruses: herpesviruses (HSV, EBV, CMV), respiratory viruses (influenza, SARS-CoV-2) Fungi: *Candida* (selected defects)	Severe recurrent bacterial, viral, and fungal infections from early childhood
Diseases of immune dysregulation	Treg, cytotoxic T cells, NK cells	Often EBV	Autoimmunity (cytopenias, IBD), lymphoproliferation, HLH
Combined immunodeficiencies with syndromic features	T and B lymphocytes	*Encapsulated bacteria*: S. pneumoniae, H. influenzae Viruses: VZV, EBV, HSV, CMV Opportunistic fungi: *Pneumocystis jirovecii*	Recurrent infections, dysmorphic features, congenital heart/skin/bone defects, malignancy
Autoinflammatory disorders	Monocytes, neutrophils, inflammasomes	None (sterile inflammation)	Recurrent fever attacks, rashes, arthritis/serositis, risk of amyloidosis
Bone marrow failure	Hematopoietic stem cells (all blood cell lines)	*Opportunistic bacteria*: *Pseudomonas aeruginosa*, *Klebsiella pneumoniae*, *Acinetobacter baumannii*, *Staphylococcus* spp., *Streptococcus* spp. Fungi: *Candida* spp., *Aspergillus* spp., *Pneumocystis jirovecii* Viruses: CMV, HSV, VZV, EBV	Pancytopenia with anemia, neutropenia, infections, cancer predisposition
Phenocopies of IEI	Autoantibodies or somatic mutations	Pathogens as in the corresponding genetic IEI (e.g., mycobacteria, fungi, viruses)	IEI-like symptoms, typically adult onset

**Table 4:** Inborn errors of immunity associated with documented pulmonary involvement: literature-based summary. Table prepared based on the 2024 IUIS classification and organ-based clinical diagnostic reference data. Abbreviations are listed in the footnotes [3,8].

PULMONARY INVOLVEMENT	ASSOCIATED INBORN ERRORS OF IMMUNITY
Asthma	CHH, CVID, DOCK8 deficiency, SIgAD, SIgMD, STAT6 GOF
Bronchiectasis	ARHGEF1 deficiency, Bloom syndrome, CF, CGD, CHH, CVID, defects of antigen presentation, GIMAP6 deficiency, HIES, IgGSD, IL6ST partial deficiency, NCKAP1L deficiency, NFATC1 deficiency, PAD, PCD, PGM3 deficiency, PGM3 deficiency, SRP19/SRPRA deficiency, STAT6 GOF, TRAF3 haploinsufficiency
Bronchitis	CVID, DPP9 deficiency, defects of antigen presentation, IgG3 deficiency, IgG4 deficiency, SIgAD, XLA
Chronic cough & pleurisy	CVID, SIgAD, XLA
Chronic Obstructive Pulmonary Disease	NFKB1 deficiency
Granulomas	RHOH deficiency, CVID
Interstitial lung disease	AR STING-associated vasculopathy, infantile-onset (SAVI), COPA Syndrome, CTLA4 haploinsufficiency (ALPS-V), ITCH deficiency (early onset), STAT5B deficiency, STAT6 GOF
Lung abscesses	AD-HIES STAT3 deficiency (Job syndrome), AR-HIES, ZNF341 deficiency
Organizing pneumonia or bronchiolitis obliterans	CID, CVID, DGS, PAD, WAS, XLP
Pneumatoceles	AD-HIES STAT3 deficiency (Job syndrome), AR-HIES, IL6ST partial deficiency, ZNF341 deficiency, iRHOM deficiency
Pneumonia	ADA deficiency, AIOLOS deficiency, AR STING-associated vasculopathy, infantile-onset (SAVI), AT, BACH2 deficiency, CARD11 deficiency (es. *Pneumocystis jirovecii*), CGD, CID, complement deficiency, COPG1 deficiency, CRACR2A deficiency, CVID, defects of antigen presentation, DPP9 deficiency, FLT3L deficiency, GIMAP6 Deficiency, GINS4 deficiency, HELIOS deficiency, iRHOM deficiency, IKAROS deficiency, IKZF2 DN (ICHAD syndrome), IL-21 deficiency, IL6 receptor deficiency, IL6ST partial deficiency, IRF4 multimorphic mutation (early onset with *Pneumocystis jirovecii*), ITCH deficiency (early onset), Kabuki syndrome (type 1 and 2), MD2 deficiency, MHC class I deficiency, NBS, NCKAP1L deficiency, NEMO deficiency, NFAT5 haploinsufficiency, NFATC1 deficiency (early onset), NFKB1 deficiency, NFKB2 deficiency, PAD, PCD, PGM3 deficiency, PLAID, PRIM1, PU1 deficiency, RAC2 deficiency, RASGRP1 deficiency, reduced serum Ig-G2 level, SASH3 deficiency, SIgAD, SLC19A1/PCFT deficiency causing hereditary folate malabsorption, SRP19 / SRPRA deficiency, STAT5B deficiency, STAT6 GOF, TLR3 deficiency (severe pulmonary influenza), TRAF3 haploinsufficiency, TWEAK deficiency, WAS, X-linked HIGM, XLA, ZNF341 deficiency
Progressive polycystic lung disease	CCR2
Pulmonary alveolar proteinosis	CCR2, CSF2R deficiency, GATA2 deficiency, SLC7A7 deficiency
Pulmonary aspergilosis	AD-HIES STAT3 deficiency (Job syndrome), IL6ST partial deficiency
Pulmonary fibrosis	DKCA, DKCB7, HCK GOF, Hermansky–Pudlak syndrome type 2
Pulmonary hypertension	GIMAP6 deficiency, PSMB9 deficiency (G156D)
Recurrent respiratory papillomatosis	NLRP1 GOF
Susceptibility to mycobacteria	MSMD
Vasculitis of lungs with pulmonary hypertension	GIMAP6 deficiency

**Table 5:** Proposed practical guide for the diagnosis and monitoring of pulmonary complications in patients with IEIs [based on [1,42,98]].

IEI DIAGNOSTICS		REMARKS
Clinical manifestations	General symptoms: • Recurrent/problematic infections • Autoimmunity • Tumors • Allergies (especially difficult to treat) Pulmonary manifestation (especially in combination with at least one of the mentioned): • Difficult to treat/diagnosed at a young age COPD • Unexplained bronchiectasis • Interstitial lung lesions of unknown etiology • Lung abscesses • Other unusual pulmonary symptoms	
Laboratory tests	ASSESSMENT OF HUMORAL IMMUNITY: • Calculated globulin (total protein—albumin) • Protein electrophoresis—total protein and γ-globulin fraction assessment • Serum levels of major immunoglobulin classes: IgA, IgG, IgM • IgG subclass levels • Isohemagglutinin titers (anti-A, anti-B)—can be performed in every blood donation center • Specific antibody titers to previously administered vaccines (tetanus, diphtheria) • Specific antibody titers after pneumococcal vaccination (in children >2 years) • Lymphocyte phenotyping—helps determine the number of B lymphocytes ASSESSMENT OF CELLULAR IMMUNITY: • Delayed-type hypersensitivity test—tuberculin skin test (TST) • Lymphocyte transformation test (LTT, mitogen-induced proliferation) • Natural killer (NK) cell cytotoxicity assay ASSESSMENT OF PHAGOCYTIC CELLS: • Production of reactive oxygen species (ROS)—NBT test (Nitroblue Tetrazolium) • Expression of adhesion molecules—flow cytometry • Assessment of chemotaxis • Assessment of particle uptake and phagocytosis ASSESSMENT OF THE COMPLEMENT SYSTEM: • Total hemolytic complement activity: CH50 • Alternative hemolytic complement activity: AH50 • C1 esterase inhibitor deficiency • Concentration of specific complement components	Proteinogram: • allows the detection of protein loss or secondary antibody deficiency • immunoglobulins are mainly present in the γ-globulin fraction (all immunoglobulin classes) and to a lesser extent in the β-globulin fraction (IgA and IgM) • in the case of deficiencies of single immunoglobulin classes, IgG subclass deficiency, or specific antibody deficiency, the result may be normal Secondary immunodeficiencies should be excluded.
Imaging tests	• Lung ultrasound • Chest X-ray • CT/MRI scan of the lungs	• For early diagnosis of pulmonary complications • Remember the contraindications for X-rays and CT scans in radiosensitivity • Consider baseline HRCT after transferring a pediatric patient to an adult center
Other	• Blood oxygen saturation • Blood gas analysis • Spirometry • Diffusing capacity for carbon monoxide (DLCO) • Bronchoscopy	• Indications determined individually (depending, among other things, on clinical manifestations) • Frequency determined individually
PULMONARY COMPLICATIONS MONITORING		REMARKS
Clinical indicators	• Chronic cough • Sputum expectoration • Dyspnea • Decreased exercise tolerance • Hemoptysis	
Laboratory tests	• Complete blood count with smear • Arterial blood gas • CRP • IgG concentration (especially in the case of supplementation)	Indications and frequency determined individually
Imaging tests	• Lung ultrasound • Chest X-ray • CT/MRI scan of the lungs	• Remember the contraindications for X-rays and CT scans in radiosensitivity • Chest X-ray once a year • HRCT at least once every 3–5 years
Microbiological testing	• Antibodies • PCR test • Swabs	• Check for any disruption in antibody production or whether the patient is taking IgTR. • Infections often cause exacerbation or worsen the course of lung disease
Other	• Arterial blood gas • Blood oxygen saturation • Spirometry • Bronchoscopy	• Frequency determined individually

## Data Availability

No new data were created or analyzed in this study. Data sharing is not applicable to this article.

## Abbreviations

The following abbreviations are used in this manuscript:

ACWY	Meningococcal conjugate vaccine (serogroups A, C, W, Y)
ADA	Adenosine Deaminase Deficiency
AD-HIES	Autosomal Dominant Hyper-IgE Syndrome (Job syndrome)
AH50	Alternative Hemolytic Complement Activity
AIOLOS	IKZF3, member of the IKAROS family of zinc-finger proteins
ALPS-V	Autoimmune Lymphoproliferative Syndrome, type V (CTLA4 haploinsufficiency)
AR	Autosomal Recessive
AR-HIES	Autosomal Recessive Hyper-IgE Syndrome
ARHGEF1	Rho Guanine Nucleotide Exchange Factor 1
AT	Ataxia-Telangiectasia
BACH2	BTB Domain And CNC Homolog 2
BTB Domain	Broad-Complex, Tramtrack, Bric-a-brac Domain (protein–protein interaction)
BTK	Bruton Tyrosine Kinase
CARD11	Caspase Recruitment Domain Family Member 11
CCR2	C-C Chemokine Receptor 2
CD3/CD4/CD8/CD19/CD16/56	Cluster of Differentiation cell markers (lymphocyte subsets)
CF	Cystic Fibrosis
CH50	Total Hemolytic Complement Activity
CHH	Cartilage–Hair Hypoplasia
CID	Combined Immunodeficiency
CNC	Cap’n’collar (transcription factor domain family)
CMV	Cytomegalovirus
CGD	Chronic Granulomatous Disease
COPA	Coatomer Protein Complex Subunit Alpha
COPG1	Coatomer Protein Complex Subunit Gamma-1
COVID-19	Coronavirus Disease 2019
CRACR2A	Calcium Release Activated Channel Regulator 2A
CSF2R	Colony Stimulating Factor 2 Receptor
CTLA4	Cytotoxic T-Lymphocyte Antigen 4
CVID	Common Variable Immunodeficiency
DGS	DiGeorge Syndrome
DKCA/DKCB7	Proteins Related to Dyskeratosis Congenita
DN	Dominant Negative
DOAJ	Directory of Open Access Journals
DOCK8	Dedicator of Cytokinesis 8
DPP9	Dipeptidyl Peptidase 9
EBV	Epstein–Barr Virus
Es.	Especially
FLT3L	FMS-like Tyrosine Kinase 3 Ligand
GATA	Guanine/Adenine/Thymine/Adenine motif
GATA2	GATA Binding Protein 2
GIMAP6	GTPase of Immunity-Associated Protein 6
GINS	Japanese acronym go-ichi-ni-san (5-1-2-3)
GINS4	GINS Complex Subunit 4
GLILD	Granulomatous-Lymphocytic Interstitial Lung Disease
GOF	Gain-of-Function
GTP	Guanosine-5′-Triphosphate
HCK GOF	Hematopoietic Cell Kinase Gain-of-Function
HELIOS	IKZF2, member of the IKAROS family of zinc-finger proteins (ICHAD syndrome)
HIES	Hyper-IgE Syndrome
HIGM	Hyper-IgM Syndrome
HLH	Hemophagocytic Lymphohistiocytosis
HPV	Human Papillomavirus
HRCT	High-Resolution Computed Tomography
HSV	Herpes Simplex Virus
IBD	Inflammatory Bowel Disease
ICHAD	Immunodeficiency, Centromeric Instability, Facial Anomalies
IEI	Inborn Errors of Immunity
IFN-I	Type I Interferon
IgA	Immunoglobulin A
IgG	Immunoglobulin G
IgGSD	IgG Subclass Deficiency
IgM	Immunoglobulin M
IKAROS	IKZF1, member of the IKAROS family of zinc-finger proteins
IKZF1	IKAROS Family Zinc Finger 1
IKZF2	IKAROS Family Zinc Finger 2
IKZF3	IKAROS Family Zinc Finger 3
IL-21	Interleukin-21
ILD	Interstitial Lung Disease
IL6ST	Interleukin-6 Signal Transducer
IUIS	International Union of Immunological Societies
IVC	Intravenous (Immunoglobulin therapy context)
iRHOM2	Inactive Rhomboid Protein 2
ITCH	Itchy E3 Ubiquitin Protein Ligase
LD	Linear Dichroism
LRBA	Lipopolysaccharide-Responsive and Beige-Like Anchor Protein
LTT	Lymphocyte Transformation Test
MAC	*Mycobacterium* Avium Complex
MD2	Myeloid Differentiation Factor 2
MDPI	Multidisciplinary Digital Publishing Institute
MHC class I	Major Histocompatibility Complex class I
MSMD	Mendelian Susceptibility to Mycobacterial Disease
NBS	Nijmegen Breakage Syndrome
NBT	Nitroblue Tetrazolium Test
NCK	Non-Catalytic Region of Tyrosine Kinase
NCKAP1L	NCK-Associated Protein 1-Like
NEMO	NF-κB Essential Modulator
NF-κB	Nuclear Factor κB (Kappa-Light-Chain-Enhancer of Activated B Cells)
NFAT5	Nuclear Factor of Activated T Cells 5
NGS	Next-Generation Sequencing
NK	Natural Killer Cells
PCV13	13-Valent Pneumococcal Conjugate Vaccine
PCV20	20-Valent Pneumococcal Conjugate Vaccine
PID	Primary Immunodeficiency
PPSV23	23-Valent Pneumococcal Polysaccharide Vaccine
ROS	Reactive Oxygen Species
SCID	Severe Combined Immunodeficiency
STAT3	Signal Transducer and Activator of Transcription 3
Tdap	Tetanus, Diphtheria, Pertussis Vaccine
TLA	Three-Letter Acronym
TLR	Toll-Like Receptor
Treg	Regulatory T Cell
TST	Tuberculin Skin Test
VZV	Varicella Zoster Virus
XLA	X-Linked Agammaglobulinemia
